# Effects of Smokeless Tobacco on Oral Health: A Cross-Sectional Study

**DOI:** 10.7759/cureus.60391

**Published:** 2024-05-15

**Authors:** Mohammad Arif Khan, Deepti Chandra, Brijendra Singh, Piyush Gowrav, Sanjay Gupta, Tulika Rani Roy

**Affiliations:** 1 Department of Periodontology, Career Post Graduate Institute of Dental Sciences and Hospital, Lucknow, IND; 2 Department of Periodontology, Babu Banarasi Das College of Dental Sciences, Lucknow, IND; 3 Department of Periodontology, Inderprastha Dental College and Hospital, Ghaziabad, IND

**Keywords:** oral mucosal changes, periodontal pocket depth, gingival recession, gingival bleeding index, gingival index, plaque index

## Abstract

Background: Smokeless tobacco (SLT) consumption poses a significant global public health challenge because of its adverse effects on oral health. Although the detrimental impact of SLT on oral tissues is well-documented, understanding its multifaceted effects is essential for effective prevention and intervention strategies.

Objective: This study aimed to comprehensively assess the impact of SLT on oral health, focusing on various clinical parameters and their differences between placement and non-placement sites of SLT.

Methods: A cross-sectional study involving 528 habitual users of SLT was conducted. Clinical parameters included the plaque index (PI), gingival index (GI), gingival bleeding index (GBI), gingival recession (GR), and probing depth (PD). Oral mucosal changes at SLT placement sites have also been reported. Statistical analysis was performed to compare parameters between the placement and non-placement sites.

Results: The study involved 528 subjects, mostly male (82%) and aged 21-40 years (mean±SD=31.14±9.10), habitual users of SLT. Prevalent SLT types included tobacco with betel nuts/masala/gutkha (59.9%) and tobacco with lime (54.5%). Significant differences were observed between SLT placement and non-placement sites: higher gingival inflammation (GI) at placement sites (1.54±0.61 vs. 1.45±0.54, p=0.01), lower GBI at placement sites (40.0% vs. 84.3%, p=0.001), and more prevalent GR (65.7% vs. 34.3%, p=0.03) at placement sites. Probing depths ≥ 3 mm were also less frequent at placement sites (2.67±0.72) than non-placement sites (3.37±1.03, p=0.001). These results highlight the detrimental impact of SLT on periodontal health, emphasizing the need for targeted interventions among SLT users.

Conclusion: SLT use is associated with adverse effects on oral health, including GI, plaque accumulation, gingival bleeding, GR, and changes in the oral mucosa. Targeted interventions and public health policies are needed to address these issues effectively.

## Introduction

Smokeless tobacco (SLT) consumption, which is prevalent across diverse cultures globally, presents a significant public health challenge owing to its detrimental effects on oral health. Unlike combustible tobacco products, which are smoked, SLT is placed inside the mouth either by chewing or snuffing. The forms may vary, including chewing tobacco and snuff, and the overarching concern lies in the potential harm inflicted on oral tissues. The adverse impact of SLT on oral health is well-established and encompasses a broad spectrum of conditions, ranging from benign lesions to malignant neoplasms [1].

The deleterious consequences of SLT use on oral health are well-documented and encompass a spectrum of conditions, from benign lesions to malignant neoplasms. SLT use has been associated with several oral manifestations localized at the site of SLT placement [2]. These manifestations include mucosal lesions and gingival periodontal defects, such as changes in gingival blood flow, gingival inflammation (GI), gingival recession (GR), and interproximal periodontal attachment loss. Understanding the intricate relationship between SLT and oral health is crucial for effective prevention and intervention strategies, given its widespread use and the potential for devastating consequences [3].

This study aimed to investigate the multifaceted impact of SLT on oral health, shedding light on its various manifestations, underlying mechanisms, and implications for public health policies and clinical practice. Through comprehensive exploration, this research endeavors to contribute to the growing body of knowledge surrounding SLT use and to empower stakeholders to effectively address this pressing public health concern.

## Materials and methods

A total of 528 habitual users of SLT were selected from the outpatient department of Periodontology, Oral Medicine, and Radiology of the Career Post Graduate Institute of Dental Sciences and dental camps organized in schools and local community centers of Lucknow, Uttar Pradesh, India, for this cross-sectional study.

Inclusion and exclusion criteria

The inclusion criteria for this study were patients who were regular users of SLT, within the age bracket of 18-60 years, and had not undergone any periodontal treatment in the preceding six months. These criteria were established to ensure a homogeneous study population with a focus on individuals actively engaged in SLT use and within an age range in which the impact on oral health could be effectively assessed.

Conversely, exclusion criteria were defined to exclude individuals with potential confounding factors that could influence the study outcomes and individuals who habitually smoked tobacco, those who were completely edentulous, and those with concurrent health disorders such as acquired immune deficiency syndrome (AIDS), cardiovascular diseases, renal diseases, and hepatitis B or C infections. Additionally, individuals currently using or recently prescribed corticosteroids, antibiotics, or nonsteroidal anti-inflammatory drugs were excluded to minimize the interference from these medications. Pregnant or lactating females were excluded from the study to avoid any potential effects of tobacco use on maternal and fetal health.

Study design

The patient’s general information was collected, and data were collected after obtaining ethical clearance from the local ethical committee. Questions were asked about the type of SLT consumption habit, such as daily/month/years of usage.

Clinical parameters, such as the plaque index (PI), gingival index (GI) by Loe in 1967, and gingival bleeding index (GBI), were also recorded. GR was measured clinically from the gingival margin to the cementoenamel junction (CEJ) using the PD Miller classification, and probing depth (PD) was measured clinically from the base of the sulcus to the gingival margin using the UNC 15 probe. Clinically, oral mucosal changes at the site of SLT placement were recorded using a specially designed pro forma following the guidelines outlined by Axell et al. [4]. Gingival and plaque indices were recorded according to the method established by Loe [5].

Criteria for GBI

The GBI by Ainamo et al. [6] was determined by gentle probing of the orifice of the gingival crevice. If bleeding occurred within 10 seconds, a positive finding was recorded, and the number of positive sites was recorded and expressed as a percentage of the number of sites examined [6]. GR was assessed using the PD Miller classification [7].

In the study focusing on habitual users of SLT, careful attention was given to the selection of oral examination sites, specifically distinguishing between placement and non-placement sites to accurately assess the localized effects of tobacco on oral tissues. The placement sites were identified based on participants' self-reported habits of where they typically placed their SLT, usually within consistent areas such as the buccal vestibule or lower labial sulcus. These areas were confirmed through visual examination for signs of tissue change such as discoloration or lesion formation. Each identified site was meticulously recorded on individual pro formas for thorough reevaluation. Conversely, non-placement sites were selected as controls, typically on the opposite side of the mouth from where tobacco was habitually placed, ensuring these areas were similar in terms of exposure to other influences such as occlusion and oral hygiene. By comparing these control sites with the tobacco placement sites, the study aimed to delineate the specific impacts of tobacco from other variables.

The study also involved a detailed examination of soft tissue lesions at the placement sites, utilizing a classification proposed by Axell et al. [4]. This classification delineated lesions into four degrees: Degree 1 indicated superficial lesions similar in color to the surrounding mucosa with slight wrinkling; Degree 2 involved superficial, whitish, or yellowish lesions with wrinkling but no thickening; Degree 3 lesions were more severe, showing a whitish-yellowish to brown coloration with wrinkling, obvious thickening, and normal color furrows; and Degree 4 consisted of heavily wrinkled, marked white-yellowish to brown lesions. This systematic assessment helped in understanding the severity and progression of tissue damage caused by chronic SLT use, providing crucial insights into its pathological impacts.

All parameters were recorded by a single examiner to prevent intra-examiner differences, and the collected data were statistically analyzed.

## Results

A total of 528 subjects were selected based on habitual use of different forms of SLT. Among those who were taking SLT, 82% of the population were male, and 18% were female. The maximum incidence of SLT consumption was observed in the age group 21-30 years (46.40%), whereas the peak SLT consumption was observed between the ages 21 and 40 years (mean±SD=31.14± 9.10). Among the different types of SLT used, tobacco with betel nuts and masala/gutkha was the most common (59.9%), followed by tobacco with lime (54.5%), tobacco with betel leaves (25.5%), betel leaves with tobacco and gutkha/masala (22.3%), and tobacco dentifrices (16.1%). The maximum incidence of SLT consumption was 54.5% population, < 5 years; 23.9% population, 5-10 years;13.6% population, 11-20%; and 8.0% population, > 20 years (Table 1).

**Table 1:** Prevalence of different tobacco habits, including betel leaf, lime, betel nut, and dentifrices among the study participants

Habit	No. (n=528)	%
Tobacco with betel leaf		
Yes	137	25.9
No	391	74.1
Tobacco with lime		
Yes	288	54.5
No	240	45.5
Tobacco with betel nut & masala/gutkha		
Yes	316	59.8
No	212	40.2
Betel leaf with tobacco & gutkha/masala		
Yes	118	22.3
No	410	77.7
Tobacco dentifrices		
Yes	85	16.1
No	443	83.9

The mean values for GI, PI, GBI, GR, and PD (in mm) showed statistically significant differences between the placement and non-placement sites of the SLT. Clinical inflammation, as measured by the GI, was significantly higher (p=0.01) at placement sites (1.54±0.61) than at non-placement sites (1.45±0.54) of SLT. The mean difference was found to be 0.01 (±0.80). The PI was less frequent at the placement sites of SLT with adjacent lesions (mean±SD=1.47±0.55) than at the non-placement sites of SLT (mean±SD=1.48±0.55). Among various SLT habits, the proportion of subjects exhibiting a PI was lower at SLT placement sites compared to non-placement sites. However, this disparity did not achieve statistical significance (p-value=0.74), with a mean difference of 0.08 (±0.80) (Table 2).

**Table 2:** Comparative analysis of the plaque index, gingival index, and probing depth between placement and opposite sites *Significant, mm: Millimeter

Site	Plaque index (mean±SD)	Gingival index (mean±SD)	Probing depth (mean±SD) in mm
Placement	1.47±0.55	1.54±0.61	2.67±0.72
Opposite	1.48±0.55	1.45±0.54	3.37±1.03
Mean difference (95%CI)	0.01±0.80	0.08±0.80	0.70±0.79
p-value	0.74	0.01*	0.001*

The percentage of GBI was lower (p=0.001) at the placement site of SLT (40.0%) adjacent to lesions than at the opposite placement site of SLT (84.3%). Gingival bleeding was absent in about 60% of the placement sites in SLT users and 15.7% of non-placement sites in SLT users. However, among the different types of SLT habits, the proportion of subjects shows that the GBI was lower at placement sites than in non-placement sites of SLT (Table 3).

**Table 3:** Comparison of the gingival bleeding index between placement and opposite sites p=0.001 (McNemar test)

Site	Present		Absent	
	No.	%	No.	%
Placement site	211	40.0	317	60.0
Opposite site	445	84.3	83	15.7

More than half of the placement sites (65.7%) of SLT users show GR, and only 34.3% of sites had no GR at placement sites, and 71.6% of sites at non-placement sites did not. According to PD Miller classification, among the different classes of GR, Class I: GR (40.7%) was the most common, followed by Class II (19.7%) and Class III (5.3%) GR at placement sites of SLT than that at non-placement sites of SLT, and was significant (p=0.07) (Table 4).

**Table 4:** Comparison of gingival recession between placement and opposite sites

Site	Normal		Class I		Class II		Class III	
	No.	%	No.	%	No.	%	No.	%
Placement site	181	34.3	215	40.7	104	19.7	28	5.3
Opposite site	378	71.6	87	16.5	40	7.6	23	4.4
p=0.07 (Kendall’s tau test)								

Clinically, the majority of subjects had mucosal changes at placement sites of SLT, and only 19.9% (n=105) were cases at placement sites of SLT. Additionally, 80.6% of the non-placement sites of SLT had no mucosal changes. However, among the different degrees of mucosal changes, according to Axell et al. [4], 44.7% (n=236) Degree 1 mucosal lesions, 22.9% (n=121) Degree 2 lesions, and 12.5% (n=66) Degree 3 lesions were observed at placement sites of SLT. However, there was a significant (p=0.04) association between the duration of smoking and the occurrence of oral mucosal changes in SLT users (Table 5).

**Table 5:** Comparison of mucosal changes between placement and opposite sites p=0.004 (Kendall’s tau test) Degree taken from Axell et al. [4]

Site	Normal		Degree 1		Degree 2		Degree 3	
	No.	%	No.	%	No.	%		
Placement site	105	19.9	236	44.7	121	22.9	66	12.5
Opposite site	431	81.6	97	18.4	0	0.0	0	0.0

The data suggest a correlation between the duration of the medical procedure and the occurrence of mucosal changes. As the duration of the procedure increases, the percentage of patients experiencing mucosal changes also appears to rise, with the highest percentage observed in the > 20-minute category (10-15%), as shown in Table 6.

**Table 6:** Association of time (in min) of smokeless tobacco retained at placement sites with mucosal changes

No. of Patients (n=528)		
Time (in min)	Percentage (%)	Mucosal changes
5-10	50-55%	Degree 1
10-20	20-25%	Degree 2
>20	10-15%	Degree 3

PD

The percentage of PD ≥ 3 mm (mean±SD=2.67±0.72) was significantly lower in placement sites adjacent to lesions than in non-placement sites of the SLT (mean±SD=3.37±1.03). Thus, statistically significant (p-value=0.001) deeper probing depths (>3 mm) were identified at non-placement sites of SLT. However, among the different types of SLT habits, the proportion of subjects showing PD was significantly lower at placement sites of SLT than at non-placement sites of SLT. The mean difference is 0.76 (±0.79) (Table 7).

**Table 7:** Comparison of GI according to habit of smokeless tobacco mm: Millimeter, GI: Gingival index, PI: plaque index, PD: Pocket depth *Significant

Habit	No. of Patients	GI		p-value	PI		p-value	PD in mm		p-value
		Placement site	Opposite site		Placement site	Opposite site		Placement site	Opposite site	
Tobacco with betel leaf										
Yes	137	1.59±0.59	1.51±0.54	0.27	1.47±0.56	1.48±0.54	0.82	2.72±0.71	3.46±1.01	0.001*
No	391	1.44±0.53	1.46±0.55	0.43	1.47±0.54	1.47±0.54	1.00	2.67±0.73	3.35±1.03	0.001*
Tobacco with lime										
Yes	288	1.49±0.56	1.48±0.55	0.86	1.48±0.54	1.51±0.55	0.48	2.64±0.74	3.31±1.03	0.001*
No	240	1.44±0.53	1.46±0.54	0.61	1.45±0.55	1.44±0.53	0.81	2.71±0.71	3.42±1.03	0.001*
Tobacco with betel nut & masala/gutkha										
Yes	316	1.44±0.52	1.43±0.54	0.74	1.46±0.55	1.48±0.56	0.52	2.71±0.73	3.40±1.04	0.001*
No	212	1.49±0.57	1.52±0.55	0.49	1.47±0.54	1.47±0.53	0.99	2.63±0.72	3.33±1.02	0.001*
Betel leaf with tobacco & gutkha/masala										
Yes	118	1.40±0.54	1.40±0.54	1.00	1.40±0.54	1.60±0.54	0.09	2.60±0.89	3.20±0.44	0.001*
No	410	1.46±0.55	1.47±0.55	0.81	1.47±0.55	1.47±0.54	0.98	2.67±0.72	3.37±1.03	0.001*
Tobacco dentifrices										
Yes	85	1.20±0.44	1.60±0.54	0.17	1.20±0.44	1.80±0.83	0.08	2.40±0.54	3.00±0.70	0.001*
No	443	1.47±0.55	1.47±0.55	1.00	1.47±0.55	1.47±0.54	0.99	2.68±0.73	3.37±1.03	0.001*

The percentage of GBI was lower (p=0.001) at the placement site of SLT (40.0%) adjacent to lesions than that at the opposite placement site of SLT (84.3%). Gingival bleeding was absent in about 60% of the placement sites of SLT users and 15.7% of non-placement sites of SLT users. However, among the different types of SLT habits, the proportion of subjects shows that GBI was lower at placement sites than in non-placement sites of SLT (Table 8).

**Table 8:** Association between tobacco habits and the gingival bleeding index (GBI) at placement and opposite sites * Significant

Habit	No. of patients	GBI								p-value
		Placement site				Opposite site				
		Present		Absent		Present		Absent		
		No.	%	No.	%	No.	%	No.	%	
Tobacco with betel leaf										
Yes	137	51	53.7	44	46.3	87	91.6	8	8.4	0.001*
No	391	262	60.5	171	39.5	400	92.4	33	7.6	0.001*
Tobacco with lime										
Yes	288	168	64.4	93	35.6	240	92.0	21	8.0	0.001*
No	240	145	54.3	122	45.7	247	92.5	20	7.5	0.001*
Tobacco with betel nut & masala/gutkha										
Yes	316	165	57.3	123	42.7	264	91.7	24	8.3	0.001*
No	212	148	61.7	92	38.3	223	92.9	17	7.1	0.001*
Betel leaf with tobacco & gutkha/masala										
Yes	118	4	80.0	1	20.0	4	80.0	1	20.0	0.001*
No	410	309	59.1	214	40.9	483	92.4	40	7.6	0.001*
Tobacco dentifrices										
Yes	85	4	80.0	1	20.0	4	80.0	1	20.0	0.001*
No	443	309	59.1	214	40.9	483	92.4	40	7.6	0.001*

## Discussion

In the present study, among the SLT users, males (82%) are associated with the habit of SLT 4.6 times more in comparison to the females (18%), which is consistent with a growing body of research suggesting that tobacco usage is higher in males than in females in India [8].

The study observed a predominance of participants from low socioeconomic backgrounds, contributing to a higher prevalence of SLT use among males. This trend may be linked to the concentration of economic power among males as well as their susceptibility to stress. Additionally, there is a common belief that SLT aids in improving concentration during occupational tasks, consistent with the findings of Mukherjee et al. [9].

Oliver et al. further corroborated the significant association between periodontal health and low socioeconomic status [10]. In the present study, a significant association was observed between different types of SLT use and the occurrence of gingival recession (65.7%) and oral mucosal lesions at the placement sites of SLT. This finding underscores a notable correlation between SLT use and periodontal injury manifested as gingival recession and mucosal changes. Remarkably, these results align with those of a previous study by Offenbacher et al. [11] reinforcing the consistency of this association across different research investigations.

Analysis based on the duration (in years) of SLT use revealed a significant association (p=0.04) between the duration of smoking habits and the presence of gingival recession. Similarly, a significant association (p=0.04) was observed between the duration of habits and oral mucosal changes. These findings are consistent with those of a previous study conducted by Hirsch et al. [12], further supporting the notion of a direct relationship between the duration of SLT use and the development of gingival recession and oral mucosal changes.

The clinical parameter for gingival index (GI) revealed a significant difference (p=0.01) between the placement sites (1.54±0.61) and non-placement sites of SLT (1.45±0.54). Interestingly, these findings contrast with those reported by Robertson et al. [13], who did not observe a relationship between SLT placement and gingivitis. However, our results were consistent with those of Poore et al. [14]. The variations in these findings may be attributed to differences in the study populations, including oral hygiene factors, which could influence the relationship between SLT placement and gingival health.

In our study, we observed that positive plaque scores were insignificantly (p=0.74) lower with adjacent lesions at the placement sites (1.47±0.55) compared to that of non-placement sites (1.48±0.55) among different types of SLT. This suggests that SLT use may not necessarily be associated with periodontal diseases. Additionally, poor oral hygiene was not found to be related to the occurrence of mucosal lesions at SLT placement sites. These findings align with those of Robertson et al. [13] and Wolfe et al. [15].

In this study, gingival bleeding was present in approximately 40% of the placement sites and 84.3% of non-placement sites of SLT, while gingival bleeding was absent in approximately 60.0% of the placement sites and 15.7% of non-placement sites of SLT. This difference was statistically significant (p=0.001). These results were consistent with the findings of Mavropoulos et al. [16] and Dietrich et al. [17] and the mechanism of action, as explained was neurogenic Inflammation induced by activation of sensory nerves subsequent release of vasodilatory peptides from their peripheral endings, known as axon reflex. However, SLT products contain high concentrations of nitrosodiethanolamine, nitrosoproline, and other tobacco-specific nitrosamines, and the pH ranges from 5.3 to 8.9. This suggests that the irritating properties of SLT, either chemical or mechanical, may contribute to the increased inflammation at placement sites. 

While it is hypothesized that nicotine's vasoconstrictive properties impair gingival blood flow, this effect may be mitigated by the elevation in blood pressure induced by smoking, which could counteract the vasoconstrictive effects of nicotine [16]. Dietrich et al. also reported that smokers have less bleeding on probing than never-smokers, which may be attributed to nicotine content. However, this finding has not been universally reported. Bleeding on probing may be influenced by various factors, such as probing force and depth, probe diameter, and the presence of inflammation [17].

In the present study, the PD was significantly lower (p=0.001) in the placement sites (2.67±0.72) than in the non-placement sites of the SLT (3.37±1.03). Moreover, significant differences in the PD were observed among different forms of SLT chewers between the placement and non-placement sites of SLT. This difference may be attributed to an increased degree of facial gingival recession. These findings are consistent with the results of a study by Robertson et al., who demonstrated a decreased percentage of PDs ≥ 3 mm in sites adjacent to SLT-induced lesions [13].

Robertson et al. [13] and Weintraub et al. [18] reported that SLT is likely to cause chemical or mechanical injury to thin areas of the gingiva that are chronically exposed to the quid, leading to loss of marginal gingiva at sites with alveolar dehiscence. Excessive tooth brushing may also contribute to mechanical injuries at sites adjacent to the lesions. However, several authors have noted that the underlying alveolar bone at buccal sites, which are prone to the development of recession, may be thin and exhibit alveolar dehiscence [19,20]. Additionally, SLT-induced epithelial proliferation may bridge the narrow lamina propria of sites with alveolar dehiscence, further contributing to the loss of marginal gingiva [21].

The mechanism of high inflammation but shallow pocket depth at the placement site likely involves a complex interplay of local tissue responses, vascular effects, activation of matrix metalloproteinases, modulation of the host immune response, and microbiological factors associated with smoking or other habits. These mechanisms collectively contribute to tissue damage, inflammation, and periodontal breakdown despite the absence of deep periodontal pockets [21].

A significant correlation (p=0.0001) was identified between the location of SLT placement and the occurrence of oral lesions. The percentage of Degree 1, 2, and 3 mucosal changes was higher in the placement sites than in the nonplacement sites. These findings are consistent with those obtained by Offenbacher et al. [11] and are in agreement with the findings of Wray et al. [22].

The oral tissues of SLT users are exposed to high concentrations of nicotine, which has detrimental effects on local cell populations. Ryder et al. [23] and Benowitz et al. [24] reported that nicotine concentrations in gingival crevicular fluid (GCF) can be up to nearly 300 times higher than plasma nicotine concentrations in tobacco users (20 ng/mL).

However, the relationship between SLT use and destructive periodontitis remains unclear. However, there is a notable and consistent association between the localized destruction of periodontal attachment, manifested as gingival recession at the placement site of SLT, and the development of oral mucosal lesions. These findings are consistent with those reported by Robertson et al. [13] and Greer et al. [25].

Epithelial proliferation and down growth along the root, accompanied by collagen and bone destruction, represent the major structural changes evident in periodontal attachment loss and gingival recession [21]. SLT-induced mucosal lesions primarily exhibit increased epithelial thickness, subepithelial inflammation, and salivary fibrosis [12]. These alterations are responses to the chemical and/or mechanical irritating effects of SLT components, potentially triggering the release of inflammatory mediators implicated in pathological changes [14].

Currently, there is insufficient evidence to confirm a consistent association between SLT use and generalized periodontal disease. Similarly, no evidence suggests that poor oral hygiene and gingivitis predispose SLT users to oral lesions. Discrepancies in findings may stem from the variability or absence of uniformity in the study populations. Therefore, future research should include a larger study population and longitudinal studies to further investigate this association.

However, gingival inflammation was notably higher at the placement sites, suggesting a localized effect of SLT use. The evident clinical alterations at these sites provide compelling evidence for the potent localized action of SLT.

The limitations of the study include the study's cross-sectional design inherently restricts the ability to establish causality between SLT use and oral health outcomes. Longitudinal studies would provide more evidence of the temporal relationship between SLT consumption and changes in oral health parameters over time. Differences between SLT placement and non-placement sites may be challenging due to variations in users' tobacco placement habits, necessitating standardized assessment methods to enhance the reliability of site-specific analyses. These limitations in future studies would contribute to a more comprehensive understanding of the impact of SLT on oral health and inform more targeted prevention and intervention strategies.

## Conclusions

Our study found significant differences between the placement site and the site opposite SLT in terms of GI, PI, GBI, GR, PD, and prevalence of oral mucosal changes. GI was higher at the placement site, whereas PI showed insignificant differences. The GBI score was lower at the placement site. The prevalence of gingival recession was higher at the placement site, and PD was lower. Oral mucosal changes were more prevalent at the placement site. Smokeless tobacco use was correlated with a higher prevalence of gingival recession, GI, and oral mucosal changes at the placement site than at the opposite site, although it was not associated with the presence of periodontal disease.
